# Amyloid-Related Imaging Abnormalities and Other MRI Findings in a Cognitively Unimpaired Population With and Without Cerebral Amyloid

**DOI:** 10.14283/jpad.2022.56

**Published:** 2022

**Authors:** R. Yaari, K.C. Holdridge, J. Choi, M.C. Donohue, K. Kantarci, C.R. Jack, S.M. Zuk, J.R. Sims, K.A. Johnson, P.S. Aisen, R.A. Sperling, A4 Study Team

**Affiliations:** 1. Eli Lilly and Company, Indianapolis, IN, USA; 2. University of Southern California, San Diego, CA, USA; 3. Mayo Clinic and Foundation, Rochester, MN, USA; 4. Massachusetts General Hospital, Boston, MA, USA; 5. Brigham and Women’s Hospital, Boston, MA, USA; 6. https://a4study.org/a4-study-team/

**Keywords:** Amyloid-related imaging abnormalities, preclinical Alzheimer’s disease, magnetic resonance imaging, florbetapir positron emission tomography

## Abstract

**BACKGROUND:**

Screening data from the Anti-Amyloid Treatment in Asymptomatic Alzheimer’s Disease (A4) and Longitudinal Evaluation of Amyloid Risk and Neurodegeneration (LEARN) studies provide a unique opportunity to compare magnetic resonance imaging (MRI) findings such as amyloid-related imaging abnormalities (ARIA) in cognitively unimpaired elderly with and without elevated cerebral amyloid.

**OBJECTIVES:**

To compare screening MRI findings, such as ARIA, in the cognitively unimpaired potential participants of a clinical trial with and without elevated cerebral amyloid.

**DESIGN:**

Cross-sectional analysis of structural MRI findings in screening data from the A4 and LEARN studies.

**SETTING:**

The A4 Study is a multi-center international clinical trial. The LEARN Study is a multi center observational study in the United States.

**PARTICIPANTS:**

Clinically normal older adults (65–85 years) with elevated cerebral amyloid (Aβ+; n = 1250, A4) and without elevated cerebral amyloid (Aβ−; n = 538, LEARN).

**MEASUREMENTS:**

Participants underwent florbetapir positron emission tomography for Aβ+/− classification. A centrally read 3T MRI to assess for study eligibility was conducted on study qualified MRI scanners.

**RESULTS:**

No ARIA-effusions (ARIA-E) was detected on screening MRI in the Aβ+ or Aβ− cohorts. At least one ARIA-H (microhemorrhages [MCH] or superficial siderosis [SS]) was present in 18% of the Aβ+ cohort compared with 8% in Aβ− (P < 0.001). In the Aβ+ cohort, approximately 2% of screening MRIs demonstrated MCH ≥4 compared with 0% in Aβ−. The presence of two apolipoprotein E ε4 (APOEε4) alleles (vs no ε4 alleles) in the Aβ+ cohort increased the odds for presence of MCH (odds ratio [OR] = 2.03; 95% CI, 1.23 to 3.27, P = 0.004). Cortical infarctions (4% vs 0%) and subcortical infarctions (10% vs 1%) were observed at statistically significantly higher prevalence in the Aβ+ cohort compared with Aβ− (P < 0.001). Females showed reduced odds of MCH in the Aβ+ cohort by a factor of 0.63 (95% CI, 0.47 to 0.84, P = 0.002).

**CONCLUSIONS:**

ARIA-E is rare in cognitively unimpaired Aβ+ and Aβ− populations prior to anti-amyloid drug intervention. ARIA-H in Aβ+ was greater than in Aβ− populations.

## Introduction

Two types of imaging abnormalities associated with anti-amyloid immunotherapy were first observed in early phase Alzheimer’s disease (AD) dementia trials (1). One type appeared to be consistent with cerebral vasogenic edema, and the other appeared to be consistent with cerebral hemosiderin deposition. These magnetic resonance imaging (MRI) abnormalities have since been associated with multiple amyloid-modifying immunotherapies. In 2011, an academic and industry leader workgroup termed these findings amyloid-related imaging abnormalities (ARIA), with presumed shared underlying pathophysiological mechanisms related to amyloid clearance from brain vasculature or parenchyma, and categorized the two types as follows: “vasogenic edema or effusion” (ARIA-E), as seen on fluid-attenuated inversion recovery (FLAIR) MRI, and hemosiderin deposition (ARIA-H) (2). ARIA-H is subdivided into the following two categories: microhemorrhages (MCH), which are small, <10 mm signal hypointensities on T2* weighted/ gradient refocused echo (GRE) MRI scans, and superficial siderosis (SS), which occur in the subarachnoid space lining the cortical surface, ≥10 mm (3).

Although associated with amyloid-modifying therapy, ARIA is also seen in the natural history of AD, but at lower rates. In patients with AD dementia without exposure to anti amyloid therapy, ARIA-E prevalence estimates range from <0.1% to 0.8% (4–7) and ARIA-H prevalence estimates range from 9.2% to 33% (6–11). In a community-dwelling population of 60- to 79-year-old individuals, the ARIA-H prevalence was 19.8% (12) and 17% in age ≥50 (mean age 69.8), with a positive amyloid positron emission tomography (PET) standardized uptake value ratio (SUVR) (13). In population cohorts of cognitively unimpaired individuals, a positive correlation was found between SUVR on amyloid PET scan and number of MCH (10, 14). Apolipoprotein E ε4 (APOEε4) is a risk factor for both ARIA-E and ARIA-H in the sporadic AD population (2).

**Figure 1.**
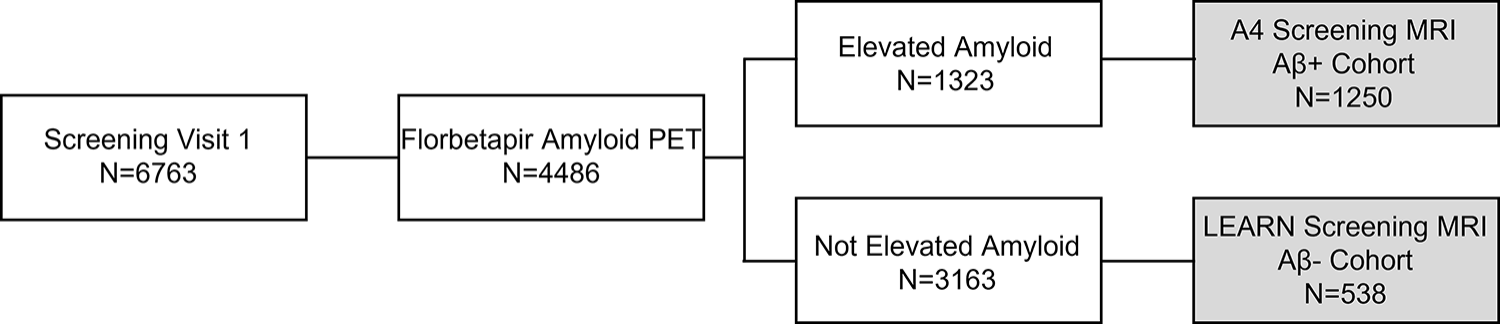
Schematic of A4 and LEARN study participants

The Anti-Amyloid Treatment in Asymptomatic AD (A4) Study is a secondary prevention trial testing solanezumab in preclinical AD (15), and the Longitudinal Evaluation of Amyloid Risk and Neurodegeneration (LEARN) Study is a companion study to A4 that enrolled individuals who met inclusion criteria for A4 on cognitive and medical measures but did not have evidence of elevated cerebral amyloid (Figure 1). Screening data from the A4 and LEARN studies allow for comparison of MRI findings including ARIA in a large cognitively unimpaired cohort both with and without elevated cerebral amyloid.

## Methods

The A4 Study (NCT02008357) is being conducted at 67 clinical trial sites in the United States, Canada, Japan, and Australia in cognitively unimpaired individuals with elevated Aβ as determined by florbetapir PET. Participants first underwent an initial clinic screening visit to assess cognitive and medical eligibility. Eligible participants then underwent florbetapir PET imaging at a second screening visit. If the PET demonstrated elevated cerebral amyloid, then a brain MRI for eligibility was conducted prior to study drug randomization in the A4 Study. If the PET did not demonstrate elevated cerebral amyloid, then participants were referred to the LEARN Study (NCT02488720), in which an MRI was also conducted for eligibility. This analysis included all screening MRIs, regardless of whether the participants were eligible to enter the study.

Participants who completed screening for the A4 and LEARN studies were aged 65 to 85 years and considered cognitively unimpaired based on a global Clinical Dementia Rating score of 0, Mini-Mental State Exam score of 25 to 30, and Logical Memory II subscale delayed paragraph recall of the Wechsler Memory Scale-Revised score of 6 to 18. Key exclusion criteria for participants were use of AD medications, unstable anxiety or depression, or other unstable medical conditions, although participants with treated hypertension, diabetes, and other common medical ailments were permitted. The 4486 participants who met these criteria then underwent florbetapir amyloid PET imaging.

### A4 Screening Amyloid PET Imaging

Florbetapir F 18 PET was acquired approximately 50 minutes after injection of 10 mCi of florbetapir F 18. Amyloid eligibility (elevated cerebral amyloid and eligible to continue in A4 screening vs not elevated and ineligible for A4 but eligible for LEARN) was assessed using an algorithm combining both quantitative SUVR methodology and qualitative visual read performed at a central laboratory. Mean SUVR using a whole cerebellar reference region of ≥1.15 was used to define elevated amyloid (Aβ+). An SUVR between 1.10 (estimated centiloid value of 24.1) and 1.15 (estimated centiloid value of 33.3) was considered to be Aβ+ only if the visual read was considered positive by consensus from two independent readers. Individuals who did not meet these amyloid PET criteria were considered not elevated or amyloid negative (Aβ−) and were eligible to screen for the LEARN Study until the target size of 500 LEARN participants was reached.

### A4 and LEARN MRI

A 3T MRI to assess for study eligibility was conducted on study qualified MRI scanners and were centrally read to help assess eligibility and describe screening MRI findings including presence of ARIA-E, ARIA-H, and evidence of other cerebral pathology. Included sequences were three-dimensional T1 MPRAGE, axial T2*/GRE, axial T2 FLAIR, axial diffusion weighted MRI, and axial T2 FSE/TSE. The A4 and LEARN studies did not have the same central readers and had slightly different variables for recording MRI findings (Appendix A). The central readers were aware of amyloid eligibility status at the time the MRIs were read.

### Statistical Analyses

Screening MRI findings from the Aβ+ and Aβ− cohorts were summarized and compared with Fisher’s exact test (binary findings) or Mann–Whitney U test (continuous or ordinal findings). The association between MRI findings and continuous potential risk factors were assessed with boxplots and Spearman’s rank correlation. The association between MRI findings and categorical variables were assessed with histograms and proportional odds models. Stepwise (forward and backward) model selection by Akaike information criterion (AIC; (16)) was used to build regression models of MRI findings with covariates for demographics and potential risk factors separately within the Aβ+ and Aβ− cohorts. Predictors considered in the model selection were as follows: age, gender, Preclinical Alzheimer’s Cognitive Composite (PACC) score, individual PACC components (Free and Cued Selective Reminding (FCSRT), Digit Symbol Substitution Test (DSST), Logical Memory IIa (LMIIa), Mini-Mental State Examination (MMSE)), Cognitive Function Index (CFI), Alzheimer’s Disease Cooperative Study-Activities of Daily Living (ADCS-ADL), number of APOE4 alleles (0,1,2), presence of APOE4 alleles (0, >0), florbetapir PET SUVr, concurrent medication of antiplatelet/antithrombotic agent (ATC2C-B01), medical history of hypertension, medical history of diabetes and medical history of both hypertension and diabetes. Note that PACC and individual components were not retained in the same final model. Logistic regression was used for binary findings and the proportional odds model for ordinal findings. Logistic regression models were checked for partial separation and infinite maximum likelihood estimates (17, 18). All analyses were conducted using R version 3.6.2.

## Results

Baseline demographics were well balanced across the Aβ+ or Aβ− cohorts (Table 1). No ARIA-E was detected on screening MRI in the Aβ+ or Aβ− cohorts (Table 1). At least one ARIA-H (MCH or SS) was present in 18% of the Aβ+ cohort compared with 8% in Aβ− (P < 0.001). In the Aβ+ cohort, approximately 2% of screening MRIs demonstrated MCH ≥4 (exclusionary for A4 Study participation) compared with 0% in Aβ−. MCH, cortical infarctions, and subcortical infarctions were observed at statistically significantly higher prevalence in the Aβ+ cohort compared with Aβ−. In the Aβ+ cohort, 11 of 18 participants with SS also had at least one MCH, whereas in the Aβ− cohort, there were no reports of SS combined with MCH. No significant correlations were observed between continuous risk factors (PACC, Total Recall, Delayed Total Recall, MMSE, Digit Symbol, CFI, ADCS-ADL, FBP PET SUVr and age in years) and number of MCH in either Aβ+ or Aβ− cohorts. The presence of two APOEε4 alleles in the Aβ+ cohort increased the odds for presence of MCH (odds ratio [OR] = 2.0267 [95% confidence interval (CI) 1.2336 to 3.2669, P = 0.004]) compared with no APOEε4 alleles (Table 2). The presence of one APOEε4 allele did not increase the odds of MCH in the Aβ+ cohort (OR = 0.9834 [95% CI, 0.7198 to 1.3464, P = 0.916]). The Aβ− cohort had two APOEε4 homozygotes—too few to analyze, but aggregating into carrier status did not increase the odds of MCH (OR = 0.726 [95% CI, 0.3064 to 1.5294, P = 0.429]). Females also showed reduced odds of MCH in the Aβ+ cohort by a factor of 0.6286 (95% CI, 0.4701 to 0.8402, P = 0.002) with equal percentages of APOEε4 carriers in both the female (425/733 = 58%) and male (312/517 = 59%) subgroups. In the Aβ+ cohort, subcortical infarction was associated with an increased odds of MCH by a factor of 2.1799 (95% CI, 1.4159 to 3.2935, P < 0.001); the Aβ− cohort did not have a sufficient number of cortical (N = 2) or subcortical infarctions (N = 3) to assess (Table 1).

A stepwise model selection (forward and backward) in the Aβ+ cohort identified that worse CFI score, greater age, and having two APOEε4 alleles increased the risk for number of MCH. Better PACC score and female sex decreased the risk for number of MCH (Table 3). Having one APOEε4 allele and history of diabetes were also in the model, but the effects were not significant. In the Aβ− cohort, no variables were identified as statistically significant predictors of MCH (Table 3). Results from stepwise model selection with MCH as a binary variable for both the Aβ+ and the Aβ− cohorts yielded similar results. The logistic regression models showed no evidence of separation or unreliable estimates due to divergence of the maximum likelihood estimates.

## Discussion

The absence of ARIA-E on screening Aβ+ MRI comports with the known low prevalence of spontaneous ARIA-E in the symptomatic AD population.

The ARIA-H prevalence in the Aβ+ cohort of 18% comports with historical data in populations with AD dementia and community-dwelling elderly populations in which amyloid status is unknown. The ARIA-H prevalence of 8% in the Aβ− cohort is lower than that in community-dwelling populations in which the amyloid status is not known, likely due to the estimate that approximately 30% of the elderly population has elevated amyloid (15, 19–21). However, cerebrovascular disease is associated with MCH, and participants with a history of substantial cerebrovascular disease were excluded from the study, which may have contributed to the lower prevalence. Additionally, despite extensive efforts to recruit a diverse population to participate in both the A4 and LEARN studies, the majority of participants were white (94% in A4 and 93% in LEARN) and non-Hispanic or Latino ethnicity (96% in both A4 and LEARN) which may have contributed to, and further reduced, the generalizability of the findings. Although the numbers were relatively small, the greater prevalence of multiple MCH (MCH ≥2) in the Aβ+ cohort compared with the Aβ− cohort also suggests a correlation of greater likelihood of multiple MCH with elevated cerebral amyloid (Aβ+ cohort [5.4%] vs Aβ− cohort [2.6%], P = 0.009). These values are comparable with multiple MCHs reported in a population-based study of non-dementia individuals ages 60 to 69 of 3%, but less than reported in older age groups: 10% aged 70 to 79, and 19% aged 80 and above (9).

The presence of a single APOEε4 allele, generally considered to be an MCH risk factor, did not appear to have a significant correlation with MCH, suggesting that a single APOEε4 allele may not be an MCH risk factor in the preclinical AD population. However, having two APOEε4 alleles approximately doubled the odds of presence of MCH in the Aβ+ cohort, and AIC selected APOEε4, but not amyloid PET SUVR. This finding is corroborated in a cohort of a community “nondemented” population that found a single APOEε4 allele to have no significant association with development of new MCHs, whereas two APOEε4 alleles had a significant association (22). In the Aβ+/− combined cohort, AIC selected both APOE and amyloid PET status, suggesting their independent predictive value. The Aβ− cohort had few APOEε4 homozygotes. Additionally, the reduced OR in females (OR = 0.6286) in the Aβ+ cohort suggests the possibility of sex-based risk of developing MCH in people who are cognitively unimpaired and Aβ+, as a greater incidence of MCH in men has been reported in a population based study of elderly adults without dementia (9). Of note, the OR for female sex in the Aβ− cohort was similar (OR = 0.6723) though not statistically significant. Use of an antiplatelet agent or anticoagulant did not significantly increase the odds of MCH in either cohort; however, a prior meta-analysis from 37 relevant studies with a sample size of 20,988 reported that MCH were more frequent in people using an antiplatelet agent (pooled OR = 1.21; 95% CI, 1.07 to 1.36; P = 0.002) (23).

In the stepwise model for predictors of MCH in the Aβ+ cohort, a better score on the PACC, a sensitive cognitive composite test, reduced odds of MCH (OR = 0.9444, P < 0.040) and an increase in CFI (indicating worsening function) increased odds of MCH (OR = 1.0728, P < 0.040), suggesting a correlation between worsened scores on clinical scales and MCH in Aβ+.

Additionally, in Aβ+, an increase in age, having two APOEε4 alleles, and history of hypertension increased odds of MCH, which has been previously reported in population studies (12). The statistically significantly reduced odds of MCH in the Aβ+ females has not previously been reported and could indicate sex differences in pathophysiology of MCH when amyloid is present.

The 1% prevalence of SS in the Aβ+ cohort is consistent with the natural history as previously reported, with an estimated prevalence of 1.43% in a community population >69 years (24). The same study reported an association between SS and increased cerebral brain amyloid SUVR, supporting CAA as a possible etiology. The difference in SS prevalence between the Aβ+ and Aβ− cohorts further supports this hypothesis. In the Aβ+ cohort, more than half of participants with SS also had MCH (11 of 18 participants); concomitant MCH were not observed in the Aβ− cohort with SS (0 of 2 participants). These results are consistent with findings in population studies: in the Mayo Clinic Study of Aging, 3 of 13 individuals with SS had concomitant MCH (24), and 7 of 7 individuals with SS had concomitant MCH in the Rotterdam Scan Study (25). These results also suggest that Aβ+ individuals have a greater likelihood for concomitant MCH and SS.

The notable elevation in screening infarction (both cortical and subcortical) in the Aβ+ cohort relative to the Aβ− cohort suggests amyloid as a risk factor for stroke, and the significantly increased odds of MCH with subcortical stroke in Aβ+ supports a common etiology, possibly due to cerebral amyloid angiopathy, which also has a strong association with APOEε4 (26). An increased risk of ischemic stroke with MCH in a community-dwelling population with unknown amyloid status has previously been reported (hazard ratio = 1.93; 95% CI, 1.25 to 2.99) (27, 28).

The prevalence of incidental cerebral infarction (combined cortical and subcortical) on MRI of 12% in the community-dwelling Lothian Birth Cohort 1936 with a mean age of 72.5 years (29) is similar to the prevalence in the Aβ+ cohort, but the prevalence in the Aβ− cohort is much lower, suggesting a correlation between cerebral infarction and elevated amyloid.

Some features of the design of the A4 and LEARN studies should be taken into consideration, which could limit conclusions from the findings. The protocol included a population of generally healthy older individuals, excluding participants with substantial vascular disease including people with significant prior infarcts or cerebral hemorrhage. In post-screening MRIs, the MRI reviewers may observe findings that were present at screening, but that could not be confidently classified due to motion or other artifacts, and therefore were not recorded as a definite finding at the time of the screening MRI. For example, the central reader may have reported no MCHs on a screening MRI, but on a subsequent MRI, the reviewer may identify an MCH that in retrospect was present at screening, and then amend the original MRI report to include the finding of an MCH; conversely, a later review could remove a previously reported finding. The A4 Study has at least four scheduled post-screening MRIs in the double-blind period, and in the LEARN Study, one scheduled post-screening MRI, which could lead to ascertainment bias.

A relatively small subset of the over 3000 Aβ− individuals from the A4 screening process underwent MRI, as the LEARN Study was only open to enroll approximately 540 individuals and finished enrollment prior to the A4 Study. Additionally, in both the A4 and LEARN studies, 3T MRIs were used, which are more sensitive to MCHs and other findings than 1.5T, which then complicates comparability to prior studies using 1.5T. Another limitation is that screening MRIs for the A4 and LEARN studies (thus, the Aβ+ and Aβ− cohorts) were reviewed by different central MRI readers with slightly different categories for reporting incidental infarctions. Although in LEARN, cortical infarcts were quantified, “subcortical infarcts” was not a LEARN variable, and for the purposes of this analysis, the free text field for “other” was reviewed to determine whether subcortical infarcts were present, which may have resulted in an underreporting of subcortical infarcts in the Aβ− cohort.

Although the screening findings of ARIA-H and incidental infarcts were higher in the Aβ+ cohort, very few of these findings met exclusion criteria for participation in the studies. Given the exploratory nature of this study, future research may further identify differences on MRI between cognitively unimpaired Aβ+ versus Aβ− populations, and associated risk factors. While also verifying the findings herein, these future studies may also help determine if risk factors for baseline ARIA-H will be risk factors for treatment emergent ARIA with anti-amyloid monoclonal antibodies, potentially providing context for safety monitoring in preclinical AD populations.

## Supplementary Material

Supplementary Material

## Figures and Tables

**Table 1.** Summary of baseline demographics and screening MRI findings in the A4 and LEARN studies

	A4 N = 1250	LEARN N = 538	Combined N = 1788	*P*-value

**Baseline Demographics**				

Age, mean (SD)	72.02 (4.85)	70.53 (4.32)	71.57 (4.75)	<0.001

Sex				0.294
Male	517 (41)	208 (39)	725 (41)	
Female	733 (59)	330 (61)	1063 (59)	

Race				0.223
American Indian or Native American	2 (0)	5 (1)	7 (0)	
Asian	27 (2)	12 (2)	39 (2)	
Black or African American	32 (3)	14 (3)	46 (3)	
White	1172 (94)	501 (93)	1673 (94)	
Multiple	9 (1)	5 (1)	14 (1)	
Unknown	8 (1)	1 (0)	9 (1)	

Ethnicity				0.835
Hispanic or Latino	37 (3)	18 (3)	55 (3)	
Not Hispanic or Latino	1201 (96)	516 (96)	1717 (96)	
Unknown	12 (1)	4 (1)	16 (1)	

Number of APOEε4 alleles				<0.001

0	511 (41)	413 (77)	924 (52)	

1	625 (50)	121 (23)	746 (42)	

2	102 (8)	2 (0)	104 (6)	

**Screening MRI Findings**				

Definite ARIA-E				
No	1250 (100)	538 (100)	1788 (100)	
Yes	0 (0)	0 (0)	0 (0)	

Definite microhemorrhage (count)				<0.001
0	1028 (82)	494 (92)	1522 (85)	
1	154 (12)	30 (6)	184 (10)	
2	35 (3)	11 (2)	46 (3)	
3	10 (1)	1 (0)	11 (1)	
≥4	23 (2)	2 (0)	25 (1)	

Definite microhemorrhage				<0.001
No	1028 (82)	494 (92)	1522 (85)	
Yes	222 (18)	44 (8)	266 (15)	

Definite superficial siderosis				0.051
No	1232 (99)	536 (100)	1768 (99)	
Yes	18 (1)	2 (0)	20 (1)	

Both definite microhemorrhage and superficial siderosis				0.041
No	1239 (99)	538 (100)	1777 (99)	
Yes	11 (1)	0 (0)	11 (1)	

≥4 definite microhemorrhage				0.014
No	1227 (98)	536 (100)	1763 (99)	
Yes	23 (2)	2 (0)	25 (1)	

Definite cortical infarction				<0.001
No	1204 (96)	536 (100)	1740 (97)	
Yes	46 (4)	2 (0)	48 (3)	

Definite subcortical infarction				<0.001
No	1131 (90)	535 (99)	1666 (93)	
Yes	119 (10)	3 (1)	122 (7)	

All data expressed as N (%) unless otherwise noted. *P*-values for screening MRI findings are from Fisher’s exact test. Participants with MRI scan date but with NA entries were considered to have "0" or "No" entries. Abbreviations: A4 = Anti-Amyloid Treatment in Asymptomatic Alzheimer’s Disease; APOEε4 = apolipoprotein E ε4; ARIA = amyloid related imaging abnormalities; ARIA-E = ARIA-effusions; LEARN = Longitudinal Evaluation of Amyloid Risk and Neurodegeneration; MRI = magnetic resonance imaging; N = number of participants; NA = not applicable; SD = standard deviation.

**Table 2.** Parameter estimates from proportional odds regression models of MCH and categorical risk factors

	A4		LEARN	

	OR (95% CI)	P-value	OR (95% CI)	*P*-value

**Number of APOEε4 alleles**				
1	0.9834 (0.7198, 1.3464)	0.9164	-	-
2	2.0267 (1.2336, 3.2669)	0.0043	-	-

**APOEε4 carrier**				
Yes	1.1084 (0.8242, 1.4972)	0.4985	0.726 (0.3064, 1.5294)	0.429

**Medical history of hypertension**				
Yes	1.2878 (0.9579, 1.7261)	0.092	1.29 (0.6914, 2.3956)	0.419

**Medical history of diabetes**				
Yes	0.6808 (0.3571, 1.1988)	0.2097	1.8765 (0.7333, 4.2154)	0.152

**Concurrent antithrombotic medication** ^**1**^				
Yes	1.0924 (0.8169, 1.4649)	0.5524	1.7963 (0.9663, 3.4083)	0.067

**Sex**				
Female	0.6286 (0.4701, 0.8402)	0.0017	0.6723 (0.3618, 1.2553)	0.208

**Definite cortical infarction**				
Yes	0.6703 (0.2532, 1.4802)	0.3662	-	-

**Definite subcortical infarction**				
Yes	2.1799 (1.4159, 3.2935)	0.0003	-	-

1 Antithrombotic agents defined as ATC-B01 = Anatomical Therapeutic Chemical Classification System Code, such as vitamin K antagonists, platelet aggregation inhibitors, and Direct factor Xa inhibitors. Abbreviations: A4 = Anti-Amyloid Treatment in Asymptomatic Alzheimer’s Disease; APOEε4 = apolipoprotein E ε4; CI = confidence interval; LEARN = Longitudinal Evaluation of Amyloid Risk and Neurodegeneration; MCH = microhemorrhage; OR = odds ratio.

**Table 3.** Predictors for number of MCH ordinal and binary in a stepwise model selection (forward and backward) in Aβ+ and Aβ-cohorts

	OR (95% CI)	*P*-value

**(A) A4 ordinal**		
PACC	0.9444 (0.8943, 0.9975)	0.040
CFI	1.0728 (1.0023, 1.1462)	0.040
Age in years	1.0431 (1.01, 1.0772)	0.010
Number of APOEε4 alleles = 1	1.0223 (0.7419, 1.4122)	0.893
Number of APOEε4 alleles = 2	2.2374 (1.3363, 3.6821)	0.002
History of diabetes	0.5967 (0.3086, 1.0684)	0.101
Sex = female	0.6878 (0.5071, 0.933)	0.016

**(B) A4 binary**		
PACC	0.9467 (0.8965, 0.9999)	0.049
CFI	1.0684 (0.9981, 1.1417)	0.053
Age in years	1.0431 (1.0097, 1.0776)	0.011
Number of APOEε4 alleles = 1	1.0352 (0.7494, 1.4335)	0.834
Number of APOEε4 alleles = 2	2.2253 (1.3238, 3.6833)	0.002
History of diabetes	0.6074 (0.3137, 1.0897)	0.114
Sex = female	0.6804 (0.5007, 0.9247)	0.014

**(C) LEARN ordinal**		
FBP PET SUVR	0.0112 (0.0001, 1.0842)	0.056
Concurrent medication of antithrombotic medication	1.764 (0.9466, 3.3543)	0.077

**(D) LEARN binary**		
FBP PET SUVR	0.0105 (0.0001, 1.0266)	0.053
Concurrent medication of antithrombotic medication	1.7993 (0.9648, 3.4237)	0.067

Notes: Variables included in the model were age, gender, PACC, CFI, ADCS-ADL, number of APOEε4 alleles (0, 1, 2), presence of APOEε4 alleles (0, >0), florbetapir PET SUVR, medical history of hypertension, concurrent medication of antiplatelet/antithrombotic agent, and medical history of diabetes. Abbreviations: A4 = Anti-Amyloid Treatment in Asymptomatic Alzheimer’s Disease; Aβ+ = elevated amyloid; Aβ- = amyloid negative; ADCS-ADL = Alzheimer’s Disease Cooperative Study—Activities of Daily Living; APOEε4 = apolipoprotein E ε4; CFI = cognitive function index; CI = confidence interval; FBP = florbetapir; LEARN = Longitudinal Evaluation of Amyloid Risk and Neurodegeneration; OR = odds ratio; PACC = Preclinical Alzheimer’s Cognitive Composite; PET = positron emission tomography; SUVR = standard uptake value ratio.
