# Supplementary material for: Amyloid-Related Imaging Abnormalities and Other MRI Findings in a Cognitively Unimpaired Population With and Without Cerebral Amyloid
Source: J Prev Alzheimers Dis. Author manuscript; Available in PMC 2024 Mar 27. (PMC10966506; doi:10.14283/jpad.2022.56)
Supplement: Supplementary Material [file NIHMS1967327-supplement-Supplementary_Material.docx]

**Appendix A.** Central reader variables for recording brain MRI findings in the A4 and LEARN studies

| **A4 Study** | **LEARN Study** |
| --- | --- |
| Definite ARIA-E (ARIAEDEF; 0 or 1) | Cerebral edema (CEDEMA; Yes/No) |
| Definite microhemorrhage (MCHDEF; 0,1,2,3,≥4) | Microhemorrhage (MICROHEM; 0,1,2,3,≥4) |
| Definite superficial siderosis (SSDEF; 0,≥1) | Hemosiderosis (HEMOSID; Yes/No) |
| Both definite microhemorrhage and superficial siderosis (MCHDEF>0 & SSDEF>0) | CEDEMA=Yes & HEMOSID=Yes |
| >4 definite microhemorrhage (MCHDEF>4) | >4 microhemorrhages (MICROHEM ≥4) |
| Definite cortical infarction (LCIDEF+SCIDEF; >0) | Cortical infarcts (CINFARCT; Yes/No) |
| Definite subcortical infarction (SUBCIDEF; 0 to 11) | Subcortical infarcts (extracted and quantified from central report narrative) |

Abbreviations: A4 = Anti-Amyloid Treatment in Asymptomatic Alzheimer’s Disease; ARIA = amyloid related imaging abnormalities; ARIA-E = ARIA-effusions; LEARN = Longitudinal Evaluation of Amyloid Risk and Neurodegeneration; MRI = magnetic resonance imaging.
